# Knowledge, attitude, and practice towards fatty liver disease among the general population in Shanghai, China: a community-based cross-sectional study

**DOI:** 10.3389/fpubh.2026.1844298

**Published:** 2026-05-28

**Authors:** Xue Yang, Hui Chang, Limei Feng, Bin Wang

**Affiliations:** 1 Yangpu Hospital, Tongji University, Yangpu, China; 2 Handan First Hospital, Handan, China

**Keywords:** cross-sectional survey, fatty liver disease, health behavior, knowledge, attitude, and practice, public awareness, Shanghai

## Abstract

**Objective:**

Concerned by the high incidence of fatty liver disease (FLD), this cross-sectional study aims to investigate the knowledge, attitude, and practice (KAP) regarding FLD among the general population in Shanghai, China.

**Methods:**

Residents of Shanghai, China, were recruited for the cross-sectional study between October 2022 and April 2023. All participants were asked to complete and submit a self-administered online questionnaire involving sociodemographic information and KAP towards FLD. A convenience sampling strategy was adopted.

**Results:**

A total of 1,006 questionnaires were included for analysis. The average scores for knowledge, attitude, and practice were 7.06 ± 2.26 (out of 13), 40.02 ± 5.70 (out of 50), and 31.09 ± 6.85 (out of 50), respectively. Correlation analysis revealed a significant correlation between attitude and practice (*r* = 0.41, *p* < 0.001). Structural equation modeling revealed that knowledge directly influenced attitude (*β* = 0.33, *p* < 0.001), and attitude influenced practice (*β* = 0.56, *p* < 0.001). In addition, knowledge indirectly influenced practice (*β* = 0.19, *p* < 0.001).

**Conclusion:**

This study shows that residents in Shanghai had inadequate knowledge, positive attitudes, and moderate practices toward FLD. These findings suggest that improving public awareness may contribute to more favorable attitudes and preventive behaviors related to FLD. Community-based health education programs, physician counseling, and digital health promotion strategies such as social media campaigns may help enhance public awareness and encourage healthier lifestyle behaviors. However, the cross-sectional design and convenience sampling may limit the generalizability of the findings.

## Background

Fatty liver disease (FLD) encompasses a group of disorders characterized by the excessive deposition of fat in the liver (1). It is generally defined as a condition where triglycerides accumulate in more than 5% of hepatocytes, regardless of the presence or absence of secondary causes, encompassing a spectrum of liver abnormalities ranging from simple steatosis to progressive liver injury (1). The two major subtypes of FLD are alcohol-related liver disease (ALD) (2), which is typically induced by excessive alcohol consumption, and nonalcoholic fatty liver disease (NAFLD) (3), defined by hepatic steatosis exceeding 5% in the absence of significant alcohol intake. Recently, a new terminology, ‘metabolic dysfunction-associated fatty liver disease’ (MAFLD), has been proposed to replace NAFLD, emphasizing the critical role of systemic metabolic dysregulation in the pathogenesis of FLD (4, 5). Currently, MASLD (formerly referred to as NAFLD/MAFLD) is recognized as a multisystemic condition that frequently coexists with type 2 diabetes mellitus, dyslipidemia, cardiovascular disease, and chronic kidney disease (6, 7). The transition from NAFLD to the MASLD nomenclature is more than a mere linguistic revision; it reflects a clinical paradigm shift toward a metabolic-centric diagnostic framework. By explicitly associating hepatic steatosis with identifiable metabolic risk factors—such as obesity and insulin resistance—this classification improves risk stratification and highlights the disease’s role as a precursor to severe extrahepatic complications. From a public health standpoint, this nomenclature update underscores the significance of modifiable metabolic determinants, laying a more solid foundation for targeted lifestyle interventions and early screening strategies that aim to alleviate the global burden of metabolic and cardiovascular morbidity.

While FLD often presents no or very mild symptoms in its early stages (8), it can progress to severe complications, which can ultimately be fatal (9). Specifically, excessive fat accumulation leads to liver cell damage and chronic inflammation, which can potentially progress to liver fibrosis, cirrhosis, or liver failure over time (10–12). FLD can also complicate other liver conditions, such as chronic hepatitis B and C, and increases the incidence of end-stage liver disease in patients with autoimmune liver diseases (13–16). Additionally, FLD is strongly associated with poor prognoses of its common comorbidities, including diabetes mellitus, cardiovascular disease, and chronic kidney disease (17–20), posing significant threats to human health. FLD is bringing an increasingly heavy public health burden as its incidence rises due to changes in lifestyle and dietary habits. It was estimated that the global prevalence of NAFLD has boosted from 25.3% in 1990–2006 to 38.0% in 2016–2019 (21). Similar trends can be observed in the US (22), Europe (23), Australia (24), and other nations. Although there is no large-scale national epidemiological research on FLD in China, prediction models show that by 2030, over 300 million Chinese individuals may suffer from FLD at various degrees (25). Recent studies also suggest that the prevalence of metabolic-related liver disease is increasing rapidly in urban areas of China, largely driven by sedentary lifestyles, dietary changes, and increasing rates of obesity and metabolic disorders. These trends highlight the growing burden of FLD in rapidly urbanizing metropolitan regions such as Shanghai. Therefore, it is crucial to recognize the severe impact of FLD and to take urgent and effective countermeasures. Unfortunately, there is a lack of quantitative data on the general population’s awareness and practices regarding FLD, making it difficult to design and implement effective preventive health policies.

The Knowledge, Attitude, Practice (KAP) theory, first introduced in the 1960s and initially applied in family planning research (26, 27), serves as a quantitative framework for studying the conceptual and behavioral characteristics of a population towards a particular issue or topic. The KAP model emphasizes the interactive relationship between knowledge, attitude, and practice and quantitatively evaluates the gap between thoughts and behaviors. Originally designed to improve public understanding, attitudes, and behaviors regarding health, education, and social issues (28, 29), KAP has evolved and has been broadly adopted in various fields, such as contraception (30), environmental protection (31), and healthcare (32), with the aim of gaining deeper insights into and influencing human behavior. In the medical context, KAP helps researchers and practitioners understand and explain patient behavior, facilitating the development of interventions to promote healthy behaviors. Previous studies conducted in several countries have reported insufficient awareness and understanding of liver diseases among the general public. For example, studies in Singapore and Malaysia found that the public had limited knowledge of liver diseases and related preventive measures (33, 34). Similarly, research conducted in the United States reported poor awareness of liver disease among adults with NAFLD (35). Other population-based investigations have also indicated that misconceptions about risk factors, disease progression, and preventive behaviors remain common in different regions, including China and other Asian countries (36–39). These findings suggest that inadequate public awareness of fatty liver disease is a global public health concern. However, many of these studies mainly focused on awareness or knowledge alone and did not comprehensively explore the interrelationships among knowledge, attitudes, and practices using the KAP framework. Moreover, community-based studies examining these relationships in the general population of mainland China remain limited. In particular, limited evidence is available regarding how knowledge of FLD may influence individuals’ attitudes toward disease prevention and subsequently affect preventive behaviors in the general population. Clarifying these pathways is essential for designing effective public health education and intervention strategies. Therefore, further research is needed to better understand the KAP status related to FLD and the pathways through which knowledge may influence attitudes and preventive behaviors.

Shanghai was selected as the study setting because it is one of the most economically developed and highly urbanized cities in China, with a large and diverse population and relatively high accessibility to healthcare services. These characteristics make Shanghai an appropriate setting for investigating public awareness and health behaviors related to chronic metabolic diseases. Moreover, urban residents are often exposed to lifestyle patterns associated with higher risks of FLD, such as sedentary behavior and dietary changes. As a major metropolitan center undergoing rapid economic development and lifestyle transitions, Shanghai provides a representative urban environment in which metabolic risk factors associated with FLD are increasingly prevalent. Investigating the KAP status of the general population in such a setting may therefore provide important insights for public health strategies targeting urban populations in China and other rapidly developing regions. Therefore, examining the general population in Shanghai may provide meaningful insights into the current status of FLD-related knowledge, attitudes, and practices in rapidly urbanizing regions of China.

This study aims to investigate the current status of knowledge, attitude, and practice towards FLD among the general population in Shanghai, China, using the KAP theoretical model, with an emphasis on the impact of knowledge on individuals’ attitudes and practices.

## Materials and methods

### Study design

This cross-sectional survey was conducted in Shanghai from October 2022 to April 2023. Participants were recruited through SMS, email, and social media (online and off-line advertisements) to complete an online questionnaire. A convenience sampling strategy was adopted, as participants were recruited through hospital-based invitations and an online survey platform rather than through probability-based random sampling. Because participants voluntarily responded to online invitations, the sample may not fully represent the general population of Shanghai, and no weighting adjustments were applied to the data. Therefore, the findings should be interpreted as reflecting the characteristics of the surveyed sample rather than the entire Shanghai population. Before participation, all respondents were informed about the purpose and procedures of the study, and electronic informed consent was obtained prior to completing the questionnaire. The study protocol was reviewed and approved by the Ethics Committee of Yangpu Hospital affiliated with Tongji University, Shanghai, China (Ethical approval No. LL-2022-SCI-009).

Inclusion criteria include (1) age of 18 years and older; (2) absence of hearing, vision, and cognitive dysfunctions; (3) ability to understand the content of the questionnaire; (4) ability to use WeChat, a Chinese social media platform, and/or email; and (5) consent to participate in the survey and signing of the informed consent form. Participants with existing liver diseases were not excluded because the purpose of the study was to assess the overall knowledge, attitudes, and practices related to fatty liver disease among the adult population. Exclusion criteria include (1) severe visual, hearing, and cognitive dysfunctions; (2) incomplete questionnaire submissions; and (3) questionnaires with uniform, malicious responses, garbled codes, or completion times under 90 s. A formal comparison between excluded and included respondents was not conducted; therefore, potential selection bias related to excluded questionnaires cannot be completely ruled out.

### Questionnaire

The questionnaire was developed with reference to the expert recommendations for the standardization of the diagnosis and treatment of FLD in China (revised in 2019) and the Nutrition and Dietary Guidelines for Chinese Residents (revised in 2022). The initial questionnaire items were drafted by the research team based on the study objectives and relevant guideline recommendations. The preliminary questionnaire was reviewed and refined by clinicians and researchers with expertise in hepatology and public health to improve its clarity, relevance, and comprehensibility.

Prior to the formal survey, a pilot study involving 51 participants was conducted to evaluate the clarity, feasibility, and reliability of the questionnaire. Based on the feedback obtained from the pilot participants, minor revisions were made to improve the wording and comprehensibility of several items. The pilot study also assessed the internal consistency of the questionnaire. The Cronbach’s alpha values for the knowledge, attitude, and behavior sections were 0.891, 0.923, and 0.868, respectively, with an overall Cronbach’s alpha of 0.936. These results indicated good internal consistency and supported the reliability of the questionnaire.

The final questionnaire, provided in Chinese, comprised 51 items across four dimensions: (1) Basic Information (18 items); (2) Knowledge (13 items); (3) Attitude (10 items); and (4) Practice (10 items). Knowledge items were scored as 1 point for correct answers and 0 points for incorrect or unclear answers, yielding a total knowledge score ranging from 0 to 13. Attitude and practice items were rated on a five-point Likert scale ranging from 1 (negative) to 5 (positive), resulting in score ranges of 10–50 for each dimension. The overall score of the questionnaire ranged from 20 to 113.

The questionnaire was distributed electronically via two pathways. In the first pathway, visitors at the medical examination center of Yangpu Hospital affiliated with Tongji University, including accompanying family members and community residents visiting the hospital, were invited to participate by scanning a QR code via WeChat. Individuals receiving treatment specifically for liver diseases were not actively recruited in order to reduce potential bias related to disease awareness and behavioral changes after diagnosis. In the second pathway, individuals aged over 18 years with IP addresses in the Shanghai area were selected from a database of 6.5 million users of Sojump,^1^ and the questionnaire was distributed through SMS, email, and platform push notifications. Considering potential differences between the hospital-based and online recruitment pathways, the two sources of respondents were recorded separately during data collection, and comparative analyses were conducted to evaluate potential bias before combining the datasets for overall analysis.

Before completing the questionnaire, all respondents were required to provide identification information, which was verified against the sample library to confirm their identities. Each respondent was allowed to participate only once, and the questionnaire was submitted after signing the embedded informed consent form. Attention check questions were included throughout the questionnaire to ensure engagement; incorrect responses rendered a questionnaire invalid. Additionally, questionnaires with uniform responses, malicious entries, garbled codes, completion times under 90 s, or abnormally low weight information were excluded.

### Sample size calculation

Based on prior studies, the incidence of FLD has been reported to range from 12.5 to 34.7%. The sample size was calculated using the formula *n* = *Z*^2^ × *p*(1 − *p*)/*δ*^2^, where *Z* = 1.96 for a two-sided *α* of 0.05, *p* = 0.25, and *δ* = 0.03. After allowing for a 15% non-response rate, the minimum required sample size was estimated to be 979. The calculation was performed using PASS version 15.0 (Power Analysis and Sample Size Software, NCSS LLC, Kaysville, Utah, USA).

### Statistical analysis

Quantitative data were described using mean ± standard deviation (SD). For data following a normal distribution, analysis of variance was employed for group comparisons, whereas non-parametric tests were used for data that did not conform to a normal distribution. Categorical data were presented as frequencies (percentages). Spearman’s correlation analysis was applied to assess the relationships among knowledge, attitude, and practice scores. Regression analysis was conducted using linear regression, and a *p*-value of less than 0.05 was considered statistically significant.

Structural equation modeling (SEM) was applied to evaluate the relationships among knowledge, attitude, and practice and to measure the indirect and direct effects between these variables. SEM was selected because it allows simultaneous assessment of complex relationships and mediating pathways among multiple variables, which is consistent with the theoretical framework of the Knowledge–Attitude–Practice model. Compared with separate regression analyses, SEM can estimate both direct and indirect effects among knowledge, attitudes, and practices within a single analytical framework.

All statistical analyses were performed using Stata version 14.0 (StataCorp LLC, College Station, TX, USA).

## Results

### Baseline information

A total of 148 and 891 valid responses were collected from the two pathways, respectively, resulting in a total of 1,006 participants included in the analysis. The participants comprised 520 males (51.69%) and 486 females (48.31%). The average body mass index (BMI) of all participants was 22.43 ± 3.19. A total of 761 participants (75.65%) were under 40 years, 684 participants (67.99%) were married, while 895 participants (88.97%) held university degrees or above. The monthly income levels varied significantly: 242 participants (24.06%) earned between 5,000 and 10,000 Chinese Yuan (CNY), 365 (36.28%) earned between 10,000 and 20,000 CNY, and 315 (31.31%) earned over 20,000 CNY.

A total of 174 individuals (17.30%) reported having liver-related diseases, among whom 107 (10.64%) had FLD. Moreover, 283 participants (18.19%) had other conditions including diabetes, hypertension, hyperlipidemia, coronary heart disease, or other related ailments, with hypertension being the most prevalent (123, 12.23%), followed by hyperlipidemia (55, 5.47%). To better contextualize the study objectives, the demographic and health characteristics of participants were first described before examining their knowledge, attitudes, and practices related to FLD. The KAP scores of respondents with and without liver diseases were further examined to explore potential differences in awareness and health behaviors related to FLD.

Most participants had at least one immediate family diagnosed with hypertension (513, 50.9%), hyperlipemia (193, 19.18%), or diabetes (240, 23.86%). Lifestyle-related information, including sleep quality, perceived stress, physical activity duration, alcohol intake, and consumption of sugar-sweetened beverages, was collected through the structured questionnaire described in the Methods section. Only 93 (9.25%) participants reported poor or very poor sleeping conditions, whereas 370 (36.78%) participants were under huge or tremendous life and work stress. In addition, most participants (811, 80.61%) spent less than 150 min on moderate-to-vigorous physical activity. More than half of the participants (670, 66.60%) consumed sugar-sweetened beverages once or more than once a day, while only 46 (4.57%) participants consumed over 15 grams of alcohol daily (Supplementary Table 1).

### Knowledge, attitude and practice distribution

To address the study objective of assessing public knowledge of FLD, the distribution of knowledge scores and responses to individual knowledge items were first analyzed. Overall, the participants showed limited knowledge of FLD. Most participants knew well about the general aspects of FLD, such as disease type (item 1), potential progression and outcome (items 2, 3, 4), transmissibility (item 7), and typical risk factors (item 8, 13). However, more than half of the participants shared common misconceptions on the clinical manifestations (items 5, 6, 10), specific risk factors (item 9), and treatment (item 11). This is not surprising because these items are more related to professional knowledge and clinical practice, which are less acknowledged by the general public.

Regarding knowledge of FLD, the respondents demonstrated varying degrees of understanding. The statement “Excessive intake of sugary drinks, heavy drinking, and lack of exercise will increase the risk of FLD” was correctly identified by 879 respondents (87.38%), indicating widespread awareness of its risk factors. Conversely, the statement “Obesity, drinking, and smoking are all risk factors for FLD” had a low accuracy rate of 4.47%, suggesting prevalent confusion or misunderstandings among the participants. A substantial majority of the respondents, 862 (85.69%), acknowledged that FLD is a common liver condition. Additionally, 818 (81.31%) were aware of its potential progression to cirrhosis. However, 310 participants (30.82%) held incorrect or unclear beliefs regarding the association between FLD and hepatocellular carcinoma (HCC).

In terms of clinical manifestations, the respondents’ knowledge appeared limited. Only 736 (73.16%) correctly understood that routine blood tests can aid in the detection of FLD. Furthermore, 797 (79.22%) were aware that the early stages of FLD could impair liver function, while 787 (78.23%) recognized symptoms such as abdominal pain and jaundice. Regarding treatment approaches, the majority, 685 respondents (68.09%), believed it is incorrect or unclear that FLD can be managed without medication. Notably, 218 (21.67%) mistakenly believed that thinner individuals are not at risk of developing FLD (Table 1).

**Table 1:** Knowledge of fatty liver disease among respondents.

Knowledge	*N* (%)	
	Wrong/unclear	Right
1. Fatty liver disease is a common type of liver disease. (True)	144 (14.31)	862 (85.69)
2. Fatty liver disease can progress to cirrhosis. (True)	188 (18.69)	818 (81.31)
3. Fatty liver disease was not associated with liver cancer. (False)	310 (30.82)	696 (69.18)
4. Once fatty liver disease is established, there is no way to return to a healthy state. (False)	354 (35.19)	652 (64.81)
5. Screening for fatty liver disease can be done by blood routine. (False)	736 (73.16)	270 (26.84)
6. Early fatty liver disease has an impact on liver function indicators such as AST/ALT. (False)	797 (79.22)	209 (20.78)
7. Fatty liver disease is not contagious. (True)	228 (22.66)	778 (77.34)
8. Excessive consumption of sugary drinks, excessive drinking, and lack of exercise can increase the likelihood of fatty liver disease. (True)	127 (12.62)	879 (87.38)
9. Obesity, alcohol and smoking are risk factors for fatty liver disease. (False)	961 (95.53)	45 (4.47)
10. Patients with fatty liver disease experience symptoms such as abdominal pain and yellow skin. (False)	787 (78.23)	219 (21.77)
11. Most patients with fatty liver disease can be treated without medication. (True)	685 (68.09)	321 (31.91)
12. Most patients with fatty liver disease require surgical treatment to have a good curative effect. (False)	439 (43.64)	567 (56.36)
13. Thinner people do not get fatty liver disease. (False)	218 (21.67)	788 (78.33)

### Attitudes toward fatty liver disease

In line with the KAP framework, respondents’ attitudes toward FLD prevention and management were subsequently examined. The vast majority of the participants demonstrated highly positive attitudes toward FLD-related topics. Specifically, over 70% of the participants showed interest or strong interest in learning and sharing FLD-related knowledge (items 1, 2, 3); nearly 80% of the participants agreed or strongly agreed that FLD required regular examination and showed great trust in their doctors, or doctors in general (items 4, 5, 6). Almost 90% of the participants held beliefs that FLD could be effectively prevented by regular exercise and a balanced diet (items 9, 10). Meanwhile, approximately half of the participants were worried about the negative consequences and serious sequelae of FLD (items 7, 8). These results again highlight the urgent need for enhanced educational programs and preventive measures tailored to increase awareness and promote proactive health behaviors in managing FLD (Table 2).

**Table 2:** Attitudes toward fatty liver disease among respondents.

Attitude	Highly agree *N* (%)	Agree *N* (%)	Neutral *N* (%)	Disagree *N* (%)	Highly disagree *N* (%)
1. You are willing to learn about fatty liver disease.	368 (36.58)	479 (47.61)	132 (13.12)	11 (1.09)	16 (1.59)
2. You are willing to participate in fatty liver related training seminars.	318 (31.61)	392 (38.97)	226 (22.47)	50 (4.97)	20 (1.99)
3. You think it’s important to spread knowledge about fatty liver disease.	449 (44.63)	405 (40.26)	120 (11.93)	22 (2.19)	10 (0.99)
4. You believe that patients with fatty liver disease need regular physical examination and review.	466 (46.32)	410 (40.76)	96 (9.54)	19 (1.89)	15 (1.49)
5. You have great trust in the treatment of fatty liver disease given by doctor.	309 (30.72)	502 (49.9)	156 (15.51)	25 (2.49)	14 (1.39)
6. You have great trust in the treatment of fatty liver disease given by your doctor.	322 (32.01)	469 (46.62)	168 (16.7)	32 (3.18)	15 (1.49)
7. You worry about cannot return to normal life after the fatty liver disease.	176 (17.5)	291 (28.93)	253 (25.15)	229 (22.76)	57 (5.67)
8. You are worried about the serious sequelae of fatty liver disease.	190 (18.89)	320 (31.81)	264 (26.24)	167 (16.6)	65 (6.46)
9. You think that maintaining moderate exercise can prevent fatty liver disease.	432 (42.94)	454 (45.13)	83 (8.25)	22 (2.19)	15 (1.49)
10. You believe that a balanced diet can prevent fatty liver disease.	480 (47.71)	422 (41.95)	68 (6.76)	23 (2.29)	13 (1.29)

### Preventive practices related to fatty liver disease

Finally, behavioral practices related to FLD prevention were analyzed to evaluate whether awareness and attitudes were reflected in preventive behaviors. The participants showed moderate engagement in practice in FLD prevention and treatment. More than half of the participants thought they would ‘always’, ‘often’, or ‘sometimes’ proactively learn knowledge about FLD (item 1), help family or friends with FLD (item 4), exercise regularly (item 5), adopt a balanced diet (item 6), consult with health professionals (item 7), and attend physical examination or FLD screening every year (item 8, 9) to prevent FLD.

In contrast, FLD does not create heavy mental burdens as most participants would not always or often suffer from anxiety or depression if they had FLD (item 3). Fewer than 20% of the participants always or often attended seminars or trainings about FLD (item 2), probably due to the limited availability of such activities. In addition, the most popular ways to learn about FLD included learning from the internet (799, 79.42%) and consulting with a specialist (690, 68.59%) (item 10) (Table 3).

**Table 3:** Practices related to fatty liver disease prevention among respondents.

Practice	Always *N* (%)	Often *N* (%)	Sometimes *N* (%)	Seldom *N* (%)	Never *N* (%)
1. You would proactively learn about fatty liver disease.	225 (22.37)	181 (17.99)	288 (28.63)	312 (31.01)	
2. You would proactively participate in seminars and trainings about fatty liver disease.	80 (7.95)	120 (11.93)	210 (20.87)	257 (25.55)	339 (33.7)
3. If you have fatty liver disease, you will suffer from anxiety, depression and other negative emotions.	86 (8.55)	210 (20.87)	310 (30.82)	258 (25.65)	142 (14.12)
4. If your family members or friends are suffering from fatty liver disease, you will share with them positive mental attitude and treatment suggestions/experiences.	216 (21.47)	415 (41.25)	250 (24.85)	87 (8.65)	38 (3.78)
5. You exercise regularly to prevent fatty liver disease.	182 (18.09)	330 (32.8)	267 (26.54)	161 (16)	66 (6.56)
6. You pay attention to your diet to prevent fatty liver disease.	221 (21.97)	361 (35.88)	257 (25.55)	118 (11.73)	49 (4.87)
7. How often do you ask professionals for advice on diet and exercise?	129 (12.82)	225 (22.37)	291 (28.93)	208 (20.68)	153 (15.21)
8. The frequency of physical examinations you attend each year.	158 (15.71)	224 (22.27)	255 (25.35)	311 (30.91)	58 (5.77)
9. You participate in fatty liver disease screenings every year.	130 (12.92)	178 (17.69)	233 (23.16)	297 (29.52)	168 (16.7)
10. Through which channels do you want to obtain knowledge about fatty liver disease? (Multiple choice questions)					
Gain knowledge on the internet from authoritative sources	799 (79.42)				
Attend public lectures organized by the community or hospital	603 (59.94)				
Consult a specialist	690 (68.59)				
Read health magazines or professional literature	527 (52.39)				
Communicate with relatives/friends	434 (43.14)				

### Correlation analysis

To further explore the relationships among the three components of the KAP framework, correlation analyses were performed. We further performed correlation analysis of the KAP scores, revealing a significant positive relationship between attitude and practice (*p* < 0.001) (Table 4).

**Table 4:** Correlation analysis of knowledge, attitude, and practice scores.

	Knowledge	Attitude	Practice
Knowledge	1		
Attitude	0.0273 (*p* = 0.3864)	1	
Practice	0.0401 (*p* = 0.2033)	0.4136 (*p* < 0.001)	1

### Multivariate linear regression analysis

To identify independent factors associated with KAP scores, multivariate regression analyses were conducted. Variables that showed potential associations in preliminary analyses were further included in multivariate regression models to identify independent factors associated with KAP scores. Multivariate linear regression analysis was conducted to explore the relationship between demographic and lifestyle factors and knowledge scores.

The results showed that FLD-related knowledge was not significantly correlated with gender, income, education level, or occupation. However, a positive correlation was observed between knowledge scores and BMI (coefficient = 0.05, 95% CI: 0.00–0.09, *p* = 0.02). Additionally, daily alcohol consumption and weekly duration of intense exercise were negatively correlated with knowledge scores (Supplementary Table 2).

Regarding attitudes, a significant positive association was observed between knowledge scores and attitude scores (coefficient = 0.31, 95% CI: 0.16–0.45, *p* < 0.001). Subgroup analysis further indicated that age, sleep quality, and stress levels significantly influenced attitude scores, with attitude scores negatively correlated with age and positively correlated with perceived stress (Supplementary Table 3).

For practice, a strong positive relationship was observed between attitude scores and practice scores (coefficient = 0.53, 95% CI: 0.46–0.60, *p* < 0.001). Practice scores were positively associated with weekly duration of high-intensity exercise and negatively associated with poor sleep quality. Married participants showed significantly higher practice scores compared with unmarried participants (coefficient = 1.74, 95% CI: 0.69–2.79, *p* = 0.001) (Supplementary Table 4).

### Structural equation modeling

To further examine the theoretical pathways proposed in the KAP framework, structural equation modeling (SEM) was conducted to evaluate the direct and indirect relationships among knowledge, attitudes, and practices.

**Figure 1:**
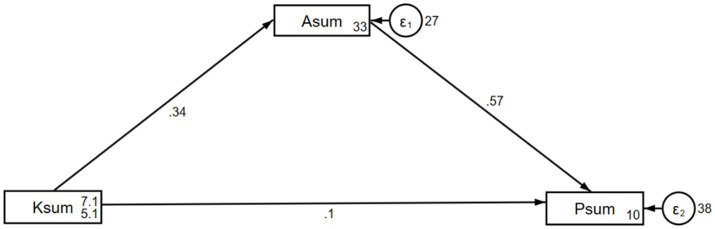
Model fit statistics of structural equation model (SEM).

The SEM goodness-of-fit analysis confirmed consistency between the observed data and the hypothesized relationships among variables (Figure 1 and Table 5). Path coefficient analysis showed that knowledge significantly influenced attitude (coefficient = 0.33, *p* < 0.001), and attitude significantly influenced practice (coefficient = 0.56, *p* < 0.001) (Table 6).

**Table 5:** Goodness-of-fit indices of the SEM.

Indicators	Reference	Results
RMSEA	<0.08 Good	0.000
SRMR	<0.08 Good	0.000
TLI	>0.8 Good	1.000
CFI	>0.8 Good	1.000

**Table 6:** Path coefficients of the SEM.

	Estimate	*p* > |*z*|
Asum<−		
Ksum	0.33	<0.001
Psum<−		
Asum	0.56	<0.001
Ksum	0.10	0.236

Further analysis of mediating effects demonstrated that knowledge had a direct effect on attitude (*β* = 0.33, 95% CI: 0.16–0.45, *p* < 0.001), while attitude had a direct effect on practice (*β* = 0.56, 95% CI: 0.49–0.64, *p* < 0.001). Although knowledge did not directly influence practice (*β* = 0.01, 95% CI: 0.00–0.27, *p* = 0.236), it significantly affected practice indirectly through attitude (*β* = 0.19, 95% CI: 0.10–0.27, *p* < 0.001). Consequently, the overall effect of knowledge on practice remained significant (*β* = 0.29, 95% CI: 0.10–0.48, *p* = 0.002) (Table 7).

**Table 7:** The direct and indirect estimates of the SEM.

Mediation effect	Total effects		Direct effect		Indirect effect	
	*β* (95% CI)	*p*	*β* (95% CI)	*p*	*β* (95% CI)	*p*
Asum<−						
Ksum	0.33 (0.19, 0.47)	<0.001	0.33 (0.19, 0.47)	<0.001	—	—
Psum<−						
Asum	0.56 (0.49, 0.64)	<0.001	0.56 (0.49, 0.64)	<0.001	—	—
Ksum	0.29 (0.10, 0.48)	0.002	0.10 (−0.0, 0.27)	0.236	0.19 (0.10, 0.27)	<0.001

## Discussion

This study on the general population of Shanghai revealed insufficient knowledge, positive attitudes, and moderate practices regarding FLD. These aspects were not significantly influenced by demographic factors such as gender, income, education, or occupation, which is consistent with prior studies (40–42). Notably, our results revealed that BMI, alcohol consumption, and duration of intense exercise were significantly correlated with knowledge; age, sleep quality, and stress levels were significantly correlated with attitudes; whereas sleep quality, duration of intense exercise, and marital status were significantly correlated with practices. SEM analysis further demonstrated that knowledge significantly influenced attitudes, while attitudes subsequently influenced practices, and practices were indirectly affected by knowledge through attitudes. These findings further support the conceptual framework of the Knowledge–Attitude–Practice (KAP) model. However, it should be noted that the observed relationships reflect statistical associations rather than causal effects due to the cross-sectional design of the study. This pathway indicates that knowledge may first shape individuals’ perceptions and beliefs about disease severity and susceptibility, which subsequently influence their willingness to adopt preventive behaviors. Therefore, improving disease awareness may contribute to healthier lifestyle choices through changes in attitudes and risk perception. Through these investigations, this study provides important insights for designing future educational and behavioral interventions for preventing and managing FLD.

Respondents demonstrated partial understanding of FLD; however, several knowledge gaps persisted, echoing findings from previous studies (33, 34). While they recognized FLD as a prevalent liver condition that could progress to cirrhosis, their understanding of its association with liver cancer was limited (35–37). Most respondents were also unaware of the symptoms and screening methods for FLD, consistent with findings from other research (38, 39). Additionally, there was confusion regarding treatment options, with many incorrectly believing that medication and surgery were the primary therapies. This aspect of knowledge has been explored only to a limited extent in prior research. Similar knowledge gaps have also been reported in population-based studies conducted in other countries, suggesting that insufficient public awareness of fatty liver disease is a widespread public health challenge rather than a problem limited to a single region. Comparable patterns of limited disease awareness have also been reported in studies of other chronic conditions, such as diabetes and cardiovascular diseases, where insufficient health literacy often affects preventive behaviors and health-seeking practices. One possible explanation is that FLD is often asymptomatic in its early stages and is frequently perceived by the public as a relatively mild or reversible condition, which may reduce the motivation to actively seek disease-related information. In addition, public health education campaigns for FLD are relatively limited compared with other chronic diseases such as diabetes or cardiovascular disease, which may further contribute to insufficient public understanding of the disease. These findings collectively underscore the need for enhanced public education about FLD.

It should be noted that the majority of participants exhibited a strong desire to acquire knowledge about FLD, highlighting the importance of public education. They acknowledged maintaining a balanced diet and engaging in moderate exercise as crucial preventive measures of FLD (43, 44). Furthermore, numerous individuals expressed concern about the potential severe consequences of the disease, fearing an inability to resume a normal lifestyle, which may be linked to insufficient understanding of the disease. Notably, knowledge scores showed a significant decline with increasing age, which may be explained by the relatively limited access of older individuals to online health information resources. Older adults may rely more on traditional sources of information and may have fewer opportunities to obtain updated health knowledge through digital platforms, which could partially explain the lower knowledge levels observed in this group. Conversely, the attitude scores of respondents increased with higher perceived stress levels. Previous research has mainly focused on the association between energy intake and nonalcoholic FLD, with limited attention given to the role of psychosocial stress. Our findings therefore suggest that psychological and lifestyle-related factors may also play an important role in shaping public perceptions and attitudes toward FLD. Individuals experiencing higher stress levels may be more sensitive to health risks and therefore more concerned about potential disease outcomes, which may lead to more cautious attitudes toward disease prevention. In addition, urban lifestyle patterns in large metropolitan areas such as Shanghai—characterized by sedentary occupations, high work pressure, and irregular dietary habits—may also influence individuals’ perceptions of metabolic diseases and their willingness to engage in preventive health behaviors. This highlights the need to consider stress and mental health factors in future research and health promotion strategies (45).

Our study revealed that the majority of respondents preferred to maintain regular exercise and focus on dietary balance to prevent FLD. However, only a minority engaged in regular disease screening, a pattern also noted in previous studies (43, 44). The association between marital status and preventive practices observed in this study may be related to differences in social support and health responsibility among married individuals. Married adults may receive greater encouragement from family members to adopt healthier lifestyles or seek medical advice, which could contribute to better preventive behaviors. Additionally, poorer sleep quality was correlated with worse practice scores, aligning with scientific findings that sleep and liver disease are closely interconnected (46). Poor sleep may also reflect broader lifestyle problems, such as irregular daily routines or increased psychological stress, which may reduce individuals’ motivation or ability to maintain healthy behaviors. In contrast, longer durations of high-intensity exercise were associated with higher practice scores, which is consistent with multiple studies showing that regular physical activity can improve metabolic health and reduce the risk of FLD (47, 48). Finally, participants tended to acquire FLD-related knowledge through diverse channels, including online resources, lectures, and consultations with professionals, suggesting that public health education strategies should adopt multiple communication approaches to reach different population groups, consistent with previous studies (49). For example, digital health education through social media platforms, physician-led counseling during routine medical visits, and community-based health promotion programs may serve as practical approaches to improve public awareness and encourage preventive behaviors related to FLD.

Notably, a positive correlation was observed between knowledge and attitudes, as well as between attitudes and practices (50). While knowledge did not exert a direct influence on practices, it indirectly affected practices by shaping attitudes. As an integral component of cognition, attitudes are strongly influenced by knowledge and contribute to the formation of sustained behavioral patterns (51). This finding highlights the importance of not only increasing disease-related knowledge but also strengthening individuals’ perception of disease risk and benefits of prevention in order to translate knowledge into sustained behavioral change. Nevertheless, improving knowledge alone may not be sufficient to ensure behavioral change, as preventive practices are also influenced by social, cultural, and environmental factors. These findings reinforce the theoretical importance of the KAP model in understanding health behaviors and suggest that multidimensional health promotion strategies combining education, clinical guidance, and community engagement may help support healthier lifestyles and preventive practices among the general population.

## Limitations

Our study has several limitations. Firstly, the study was conducted in Shanghai, a highly developed urban region with relatively advanced healthcare resources, which may limit the generalizability of the findings to rural or less developed regions. Future studies should consider multicenter designs involving different geographic regions to provide more representative evidence. Secondly, the use of convenience sampling and online questionnaire distribution may have introduced selection bias, as individuals with higher education levels and greater access to digital technologies were more likely to participate in the survey, which may limit the representativeness of the sample. Thirdly, the proportion of individuals aged 65 years and older in our sample was relatively low, possibly due to the limited use of electronic devices among older adults. As this population group is at higher risk for metabolic disorders and fatty liver disease, future research should adopt alternative recruitment strategies to ensure better representation of older individuals. Fourthly, considering the large population base of Shanghai, the sample size of the present study remains relatively small. Fifthly, as an online questionnaire-based survey, only invalid questionnaires were eliminated after rigorous evaluation of response quality. Moreover, no pre-specified subgroup analysis regarding different categories of liver diseases was formulated during the questionnaire design period. In-depth investigations focusing on specific populations will be carried out in future studies. Finally, although efforts were made to recruit participants from the general population, part of the sample was collected through hospital-based recruitment, which may introduce potential selection bias related to health awareness and healthcare utilization. Therefore, the findings of this study should be interpreted with caution when generalizing the results to the broader population.

## Conclusion

In conclusion, this community-based cross-sectional study revealed that residents in Shanghai generally had insufficient knowledge but relatively positive attitudes and moderate practices regarding fatty liver disease. The findings further demonstrated that knowledge influenced attitudes, and attitudes subsequently influenced preventive practices, supporting the theoretical framework of the Knowledge–Attitude–Practice model. These results highlight the importance of strengthening public education on fatty liver disease, promoting healthy lifestyles, and improving awareness of preventive strategies among the general population. Future studies should involve larger and more diverse populations to further explore effective interventions for improving public awareness and preventive behaviors related to fatty liver disease.

## Data Availability

The original contributions presented in the study are included in the article/Supplementary material, further inquiries can be directed to the corresponding authors.

## Footnotes

1
https://www.wjx.cn/
