# Supplementary material for: Knowledge, attitude, and practice towards fatty liver disease among the general population in Shanghai, China: a community-based cross-sectional study
Source: Front Public Health. 2026 May 28;14:1844298. doi: 10.3389/fpubh.2026.1844298 (PMC13255343; doi:10.3389/fpubh.2026.1844298)
Supplement: Supplementary file 1 [file Table_1.docx]

**Suppl 1 Baseline demographic and health characteristics of the participants**

| ***N*=1006** | **N (%)** | **Knowledge Score** | | **Attitude Score** | | **Practice Score** | |
| --- | --- | --- | --- | --- | --- | --- | --- |
|  |  | **Mean ± SD** | **P** | **Mean ± SD** | **P** | **Mean ± SD** | **P** |
| **Total Score** |  | 7.06 ±2.26 |  | 40.02±5.70 |  | 31.09±6.85 |  |
| **Gender** |  |  | 0.941 |  | 0.988 |  | 0.110 |
| Male | 520(51.69) | 7.09±2.18 |  | 35.67±5.27 |  | 30.80±6.78 |  |
| Female | 486(48.31) | 7.02±2.34 |  | 35.72±5.16 |  | 31.40±6.91 |  |
| **Age (years)** |  |  | <0.001 |  | 0.002 |  | <0.001 |
| <30 | 303(30.12) | 6.45±2.22 |  | 35.91±5.43 |  | 29.55±6.51 |  |
| 31-40 | 458(45.53) | 7.31±2.22 |  | 35.92±5.19 |  | 32.47±7.07 |  |
| 41-50 | 175(17.4) | 7.23±2.14 |  | 35.28±5.09 |  | 30.92±6.29 |  |
| 51-60 | 50(4.97) | 7.66±2.41 |  | 34.7±4.46 |  | 29.46±6.33 |  |
| >60 | 20(1.99) | 7.4±2.72 |  | 33.4±4.53 |  | 28.2±6.45 |  |
| **Marital status** |  |  | 0.003 |  | 0.003 |  | <0.001 |
| Never married | 301(29.92) | 6.63±2.35 |  | 35.50±5.51 |  | 28.81±6.38 |  |
| Married | 684(67.99) | 7.25±2.19 |  | 35.88±5.06 |  | 32.21±6.77 |  |
| Divorced | 16(1.59) | 6.5±2.55 |  | 33.37±5.27 |  | 26.5±7.05 |  |
| Widowed | 5(0.5) | 8±1.22 |  | 29.4±2.88 |  | 29.2±5.63 |  |
| **Highest degree** |  |  | <0.001 |  | 0.055 |  | 0.032 |
| Junior high school and below | 18(1.79) | 7.05±1.83 |  | 35.11±4.25 |  | 28.38±7.27 |  |
| Senior high school | 93(9.24) | 6.40±2.23 |  | 35.72±5.39 |  | 29.88±7.21 |  |
| University | 758(75.35) | 7.02±2.21 |  | 35.52±5.29 |  | 31.15±6.76 |  |
| Postgraduate or above | 137(13.62) | 7.70±2.42 |  | 36.71±4.71 |  | 31.90±6.93 |  |
| **Monthly household income (including physical income, rental income, etc.)** |  |  | <0.001 |  | 0.369 |  | <0.001 |
| <2000 CNY | 12(1.19) | 6.83±2.12 |  | 36.41±6.14 |  | 29.58±5.79 |  |
| 2000-5000 CNY | 72(7.16) | 6.19±2.54 |  | 35.04±6.84 |  | 28.43±7.32 |  |
| 5000-10000 CNY | 242(24.06) | 6.83±1.93 |  | 35.36±5.30 |  | 30.30±7.06 |  |
| 10000-20000 CNY | 365(36.28) | 7.10±2.38 |  | 35.58±5.11 |  | 31.28±6.67 |  |
| >20000 CNY | 315(31.31) | 7.39±2.23 |  | 36.21±4.78 |  | 32.13±6.62 |  |
| **Occupation type** |  |  | <0.001 |  | 0.020 |  | <0.001 |
| Leader of governemental organizations, enterprises or institutions | 101(10.04) | 6.86±1.93 |  | 36.42±4.32 |  | 35.03±6.35 |  |
| Professional and technical personnel (teachers, doctors, engineering and technical personnel, writers and other professionals) | 268(26.64) | 7.75±2.25 |  | 36.26±5.21 |  | 32.28±6.55 |  |
| Office staff and related personnel | 215(21.37) | 7.12±2.01 |  | 35.00±4.99 |  | 30.93±6.37 |  |
| Business, service personnel | 162(16.1) | 6.96±2.28 |  | 35.38±5.25 |  | 30.03±6.36 |  |
| Agricultural, forestry, animal husbandry, fishery water conservancy production personnel | 14(1.39) | 6.28±1.89 |  | 34.71±4.41 |  | 31.85±6.17 |  |
| Production, transportation equipment operators and related personnel | 49(4.87) | 6.48±2.15 |  | 36.10±4.19 |  | 31±5.70 |  |
| Military personnel | 2(0.2) | 6.5±0.70 |  | 40.5±6.36 |  | 36±7.07 |  |
| Other | 195(19.38) | 6.44±2.48 |  | 35.49±6.00 |  | 28.37±7.42 |  |
| **BMI** | 22.43±3.19 |  |  |  |  |  |  |
| **Whether you have liver disease** |  |  |  |  |  |  |  |
| Viral hepatitis (such as hepatitis B, hepatitis C) | 31(3.08) |  |  |  |  |  |  |
| Alcoholic Liver disease | 24(2.39) |  |  |  |  |  |  |
| Fatty liver disease | 107(10.64) |  |  |  |  |  |  |
| Drug-induced hepatitis | 4(0.4) |  |  |  |  |  |  |
| Autoimmune hepatitis | 7(0.7) |  |  |  |  |  |  |
| Hereditary liver disease | 2(0.2) |  |  |  |  |  |  |
| Liver cirrhosis | 5(0.5) |  |  |  |  |  |  |
| Liver cancer | / |  |  |  |  |  |  |
| Other | 13(1.29) |  |  |  |  |  |  |
| None | 832(82.7) |  |  |  |  |  |  |
| **Have any of the following medical conditions** |  |  |  |  |  |  |  |
| Diabetes | 8(0.8) |  |  |  |  |  |  |
| Hypertension | 123(12.23) |  |  |  |  |  |  |
| Hyperlipemia | 55(5.47) |  |  |  |  |  |  |
| Coronary heart disease | 7(0.7) |  |  |  |  |  |  |
| Hyperuricemia/gout | 14(1.39) |  |  |  |  |  |  |
| Other | 7(0.7) |  |  |  |  |  |  |
| None | 823(81.81) |  |  |  |  |  |  |
| **Any of the following diseases in your immediate family** |  |  |  |  |  |  |  |
| Diabetes | 240(23.86) |  |  |  |  |  |  |
| Hypertension | 513(50.99) |  |  |  |  |  |  |
| Hyperlipemia | 193(19.18) |  |  |  |  |  |  |
| Coronary heart disease | 100(9.94) |  |  |  |  |  |  |
| Fatty liver disease | 24(2.39) |  |  |  |  |  |  |
| Hyperuricemia/gout | 61(6.06) |  |  |  |  |  |  |
| Other | 11(1.09) |  |  |  |  |  |  |
| None | 368(36.58) |  |  |  |  |  |  |
| **Sleep quality** |  |  | 0.226 |  | <0.001 |  | <0.001 |
| Very good | 187(18.59) | 7.08±2.33 |  | 36.39±5.75 |  | 32.91±7.39 |  |
| Good | 410(40.76) | 7.04±2.19 |  | 35.90±5.12 |  | 31.88±6.73 |  |
| Neutral | 316(31.41) | 6.98±2.29 |  | 35.16±5.17 |  | 29.51±6.62 |  |
| Poor | 85(8.45) | 7.23±2.35 |  | 34.71±4.28 |  | 29.35±5.43 |  |
| Very poor | 8(0.8) | 8.75±1.75 |  | 40.37±2.55 |  | 28.87±5.35 |  |
| **How stressful you feel about your daily life and work** |  |  | 0.039 |  | <0.001 |  | 0.029 |
| Rarely | 51(5.07) | 6.31±2.62 |  | 33.15±7.27 |  | 30.58±7.87 |  |
| Less | 176(17.5) | 7.26±2.18 |  | 36.25±4.08 |  | 32.63±6.99 |  |
| General | 409(40.66) | 6.94±2.38 |  | 35.02±5.50 |  | 30.81±6.98 |  |
| Neutral | 333(33.1) | 7.25±2.05 |  | 36.49±4.73 |  | 30.73±6.36 |  |
| Tremendous | 37(3.68) | 6.64±2.15 |  | 36.78±5.71 |  | 30.72±6.83 |  |
| **Number of meals you eat every day** |  |  | 0.032 |  | 0.032 |  | 0.001 |
| 1-2 times | 161(16) | 6.57±2.36 |  | 34.62±6.05 |  | 29.11±6.99 |  |
| 3 times | 829(82.41) | 7.15±2.22 |  | 35.93±4.98 |  | 31.47±6.78 |  |
| 4-5 times | 16(1.59) | 6.93±2.64 |  | 34.5±6.75 |  | 31.18±5.92 |  |
| **How much snacks do you eat every day** |  |  | 0.228 |  | 0.296 |  | 0.260 |
| None | 115(1.43) | 7.06±2.52 |  | 36.06±5.70 |  | 31.16±7.21 |  |
| Less (1-2 times) | 598(59.44) | 7.17±2.16 |  | 35.88±4.98 |  | 31.19±6.76 |  |
| Neutral (3-4 times) | 199(19.78) | 6.89±2.14 |  | 35.52±4.81 |  | 30.96±6.64 |  |
| Often (5-6 times) | 85(8.45) | 6.8±2.71 |  | 34.63±6.61 |  | 31.11±7.51 |  |
| Always (more than 6 times) | 9(0.89) | 5.77±2.53 |  | 32.88±6.77 |  | 25.77±5.21 |  |
| **Your daily intake of sugary beverages** |  |  | 0.100 |  | 0.180 |  | 0.875 |
| None | 336(33.4) | 7.27±2.41 |  | 35.56±5.37 |  | 31.22±6.83 |  |
| Neutral (1-2 times) | 589(58.55) | 6.95±2.10 |  | 35.90±4.98 |  | 31.00±6.86 |  |
| More (more than 3 times) | 81(8.05) | 6.93±2.60 |  | 34.76±6.12 |  | 31.19±6.89 |  |
| **Your average weekly amount of moderate-to-vigorous physical activity** |  |  | 0.036 |  | 0.349 |  | <0.001 |
| None | 259(25.75) | 7.34±2.48 |  | 35.48±5.42 |  | 27.53±6.26 |  |
| 0-150min | 552(54.87) | 7.03±2.12 |  | 35.78±4.94 |  | 31.83±6.66 |  |
| 150-300min | 156(15.51) | 6.85±2.16 |  | 35.93±5.47 |  | 33.74±6.02 |  |
| >300min | 39(3.88) | 6.38±2.73 |  | 34.97±6.58 |  | 33.51±7.39 |  |
| **Your daily alcohol intake** |  |  | 0.004 |  | 0.978 |  | <0.001 |
| None | 734(72.96) | 7.15±2.32 |  | 35.69±5.34 |  | 30.55±7.01 |  |
| Less than 15g alcohol (15g alcohol is about 450ml beer or 150ml wine or 50g (1 two) 38 proof liquor or 30g 52 proof liquor) | 226(22.47) | 6.95±2.06 |  | 35.64±4.87 |  | 32.70±6.03 |  |
| More than 15g alcohol (including 15g) | 46(4.57) | 6.15±2.08 |  | 35.97±4.92 |  | 31.73±6.90 |  |
