# Supplementary material for: Knowledge, attitude, and practice towards fatty liver disease among the general population in Shanghai, China: a community-based cross-sectional study
Source: Front Public Health. 2026 May 28;14:1844298. doi: 10.3389/fpubh.2026.1844298 (PMC13255343; doi:10.3389/fpubh.2026.1844298)
Supplement: Supplementary file 2 [file Table_2.docx]

**Suppl 2 Multivariate linear regression analysis of factors associated with knowledge scores.**

| **Knowledge** | **Univariate analysis** | | **Multivariate analysis** | |
| --- | --- | --- | --- | --- |
|  | **Coef. (95%CI)** | **P** | **Coef. (95%CI)** | **P** |
| **Gender** |  |  |  |  |
| Male |  |  |  |  |
| Female | -0.07(-0.35,0.20) | 0.597 |  |  |
| **Age (years)** |  |  |  |  |
| <30 |  |  |  |  |
| 31-40 | 0.86(0.54,1.19) | **<0.001** | 0.62(0.21,1.04) | **0.003** |
| 41-50 | 0.78(0.36,1.19) | **<0.001** | 0.47(-0.04,0.98) | 0.072 |
| 51-60 | 1.20(0.53,1.87) | **<0.001** | 0.96(0.22,1.70) | **0.01** |
| >60 | 0.94(-0.06,1.95) | 0.066 | 0.49(-0.59,1.59) | 0.374 |
| **Marital status** |  |  |  |  |
| Never married |  |  |  |  |
| Married | 0.62(0.32,0.93) | **<0.001** | 0.00(-0.41,0.41) | 0.998 |
| Divorced | -0.13(-1.26,0.99) | 0.82 | -0.71(-1.85,0.43) | 0.223 |
| Widowed | 1.36(-0.61,3.35) | 0.177 | 1.23(-0.79,3.26) | 0.234 |
| **Highest degree** |  |  |  |  |
| Junior high school and below |  |  |  |  |
| Senior high school | -0.64(-1.78,0.48) | 0.263 |  |  |
| Bachelor | -0.03(-1.08,1.01) | 0.955 |  |  |
| Postgraduate or above | 0.65(-0.45,1.75) | 0.247 |  |  |
| **Monthly household income (including physical income, rental income, etc.)** |  |  |  |  |
| < 2000 CNY |  |  |  |  |
| 2000-5000 CNY | -0.63(-2.01,0.73) | 0.362 |  |  |
| 5000-10000 CNY | 0.00(-1.29,1.30) | 0.993 |  |  |
| 10000-20000 CNY | 0.26(-1.02,1.56) | 0.684 |  |  |
| >20000 CNY | 0.56(-0.73,1.85) | 0.396 |  |  |
| **Occupation** |  |  |  |  |
| Leader of governemental organizations, enterprises or institutions |  |  |  |  |
| Professional and technical personnel (teachers, doctors, engineering and technical personnel, writers and other professionals) | 0.88(0.38,1.39) | **0.001** | 0.70(0.19,1.21) | 0.007 |
| Office staff and related personnel | 0.25(-0.26,0.78) | 0.332 | 0.13(-0.38,0.65) | 0.617 |
| Business, service personnel | 0.10(-0.45,0.65) | 0.718 | 0.01(-0.53,0.56) | 0.963 |
| Agricultural, forestry, animal husbandry, fishery water conservancy production personnel | -0.57(-1.81,0.66) | 0.363 | -0.44(-1.70,0.80) | 0.484 |
| Production, transportation equipment operators and related personnel | -0.37(-1.12,0.38) | 0.336 | -0.41(-1.16,0.33) | 0.282 |
| Military personnel | -0.36(-3.47,2.74) | 0.82 | -0.03(-3.10,3.04) | 0.985 |
| Other | -0.42(-0.95,0.11) | 0.123 | -0.27(-0.84,0.29) | 0.344 |
| **BMI** | 0.06(0.02,0.11) | **0.002** | 0.05(0.00,0.09) | **0.02** |
| **Sleep quality** |  |  |  |  |
| Very good |  |  |  |  |
| Good | -0.03(-0.42,0.35) | 0.865 | -0.09(-0.48,0.29) | 0.626 |
| Neutral | -0.09(-0.50,0.31) | 0.635 | -0.17(-0.60,0.24) | 0.407 |
| Poor | 0.15(-0.42,0.73) | 0.6 | 0.08(-0.51,0.67) | 0.784 |
| Very poor | 1.66(0.06,3.27) | **0.041** | 1.33(-0.26,2.94) | 0.101 |
| **How stressful you feel about your daily life and work** |  |  |  |  |
| Rarely |  |  |  |  |
| Less | 0.95(0.24,1.65) | **0.008** | 0.89(0.18,1.60) | **0.014** |
| General | 0.63(-0.02,1.28) | 0.06 | 0.54(-0.12,1.22) | 0.111 |
| Neutral | 0.94(0.27,1.60) | **0.005** | 0.88(0.18,1.59) | **0.013** |
| Tremendous | 0.33(-0.62,1.29) | 0.492 | 0.11(-0.87,1.11) | 0.814 |
| **Number of meals you eat every day** |  |  |  |  |
| 1-2 times |  |  |  |  |
| 3 times | 0.58(0.19,0.96) | **0.003** | 0.28(-0.10,0.67) | 0.153 |
| 4-5 times | 0.35(-0.80,1.51) | 0.543 | 0.08(-1.06,1.24) | 0.879 |
| **How much snacks do you eat every day** |  |  |  |  |
| None |  |  |  |  |
| Less (1-2 times) | 0.11(-0.33,0.56) | 0.623 |  |  |
| Neutral (3-4 times) | -0.16(-0.68,0.35) | 0.53 |  |  |
| Often (5-6 times) | -0.26(-0.89,0.37) | 0.42 |  |  |
| Always (more than 6 times) | -1.28(-2.81,0.25) | 0.101 |  |  |
| **Your daily intake of sugary beverages** |  |  |  |  |
| None |  |  |  |  |
| Neutral (1-2 times) | -0.32(-0.62,-0.02) | **0.035** | -0.16(-0.46,0.14) | 0.309 |
| More (more than 3 times) | -0.34(-0.89,0.20) | 0.222 | -0.14(-0.70,0.42) | 0.626 |
| **Your average weekly amount of moderate-to-vigorous physical activity** |  |  |  |  |
| None |  |  |  |  |
| 0-150min | -0.30(-0.64,0.02) | 0.071 | -0.29(-0.62,0.04) | 0.09 |
| 150-300min | -0.49(-0.93,-0.04) | **0.032** | -0.64(-1.11,-0.1) | **0.006** |
| >300min | -0.95(-1.71,-0.19) | **0.013** | -0.84(-1.61,-0.0) | **0.032** |
| **Your daily alcohol intake** |  |  |  |  |
| None |  |  |  |  |
| Less than 15g alcohol (15g alcohol is about 450ml beer or 150ml wine or 50g (1 two) 38 proof liquor or 30g 52 proof liquor) | -0.20(-0.53,0.13) | 0.241 | -0.25(-0.60,0.08) | 0.143 |
| More than 15g alcohol (including 15g) | -1.00(-1.67,-0.32) | **0.004** | -1.00(-1.69,-0.31) | **0.005** |
