# Supplementary material for: Knowledge, attitude, and practice towards fatty liver disease among the general population in Shanghai, China: a community-based cross-sectional study
Source: Front Public Health. 2026 May 28;14:1844298. doi: 10.3389/fpubh.2026.1844298 (PMC13255343; doi:10.3389/fpubh.2026.1844298)
Supplement: Supplementary file 3 [file Table_3.docx]

**Suppl 3 Multivariate linear regression analysis of factors associated with attitude scores.**

| **Attitude** | **Univariate analysis** | | **Multivariate analysis** | | |
| --- | --- | --- | --- | --- | --- |
|  | **Coef. (95%CI)** | **P** | **Coef. (95%CI)** | **P** | |
| **Knowledge score** | 0.33(0.19,0.47) | **<0.001** | 0.31(0.16,0.45) | | **<0.001** |
| **Gender** |  |  |  | |  |
| male |  |  |  | |  |
| female | 0.05(-0.59,0.69) | 0.872 |  | |  |
| **Age (years)** |  |  |  | |  |
| <30 |  |  |  | |  |
| 31-40 | 0.009(-0.74,0.76) | 0.981 | -0.80(-1.76,0.15) | | 0.1 |
| 41-50 | -0.62(-1.59,0.34) | 0.204 | -1.38(-2.54,-0.22) | | **0.019** |
| 51-60 | -1.21(-2.77,0.34) | 0.127 | -1.86(-3.53,-0.19) | | **0.029** |
| >60 | -2.51(-4.87,-0.15) | **0.037** | -2.73(-5.22,-0.25) | | **0.031** |
| **Marital status** |  |  |  | |  |
| Never married |  |  |  | |  |
| Married | 0.38(-0.32,1.08) | 0.285 | 0.66(-0.27,1.60) | | 0.166 |
| Divorced | -2.12(-4.74,0.48) | 0.111 | -0.88(-3.52,1.75) | | 0.51 |
| Widowed | -6.10(-10.7,-1.50) | **0.009** | -3.98(-8.66,0.70) | | 0.096 |
| **Highest degree** |  |  |  | |  |
| Junior high school and below |  |  |  | |  |
| Senior high school | 0.60(-2.02,3.24) | 0.65 |  | |  |
| University | 0.41(-2.02,2.85) | 0.738 |  | |  |
| Postgraduate or above | 1.60(-0.95,4.16) | 0.22 |  | |  |
| **Monthly household income (including physical income, rental income, etc.)** |  |  |  | |  |
| <2000 CNY |  |  |  | |  |
| 2000-5000 CNY | -1.37(-4.56,1.81) | 0.398 |  | |  |
| 5000-10000 CNY | -1.04(-4.07,1.97) | 0.497 |  | |  |
| 10000-20000 CNY | -0.83(-3.83,2.16) | 0.585 |  | |  |
| >20000 CNY | -0.20(-3.21,2.80) | 0.894 |  | |  |
| **Occupation type** |  |  |  | |  |
| Leader of governemental organizations, enterprises or institutions |  |  |  | |  |
| Professional and technical personnel (teachers, doctors, engineering and technical personnel, writers and other professionals) | -0.16(-1.35,1.02) | 0.787 | -0.72(-1.89,0.44) | | 0.227 |
| Office staff and related personnel | -1.42(-2.65,-0.18) | **0.024** | -1.56(-2.75,-0.36) | | **0.01** |
| Business, service personnel | -1.04(-2.33,0.25) | 0.114 | -1.23(-2.50,0.02) | | 0.055 |
| Agricultural, forestry, animal husbandry, fishery water conservancy production personnel | -1.71(-4.62,1.20) | 0.249 | -0.28(-3.15,2.58) | | 0.844 |
| Production, transportation equipment operators and related personnel | -0.32(-2.10,1.45) | 0.721 | -0.28(-2.01,1.44) | | 0.747 |
| Military personnel | 4.07(-3.22,11.3) | 0.273 | 2.86(-4.22,9.95) | | 0.428 |
| Other | -0.92(-2.18,0.32) | 0.146 | -0.57(-1.88,0.72) | | 0.382 |
| **BMI** | 0.03(-0.06,0.13) | 0.495 |  | |  |
| **Sleep quality** |  |  |  | |  |
| Very good |  |  |  | |  |
| Good | -0.48(-1.38,0.41) | 0.291 | -0.86(-1.75,0.02) | | 0.057 |
| Neutral | -1.22(-2.16,-0.28) | **0.011** | -1.58(-2.55,-0.61) | | **0.001** |
| Poor | -1.67(-3.00,-0.34) | **0.014** | -2.41(-3.77,-1.05) | | **0.001** |
| Very poor | 3.98(0.30,7.65) | **0.034** | 2.32(-1.35,6.01) | | 0.215 |
| **How stressful you feel about your daily life and work** |  |  |  | |  |
| Rarely |  |  |  | |  |
| Less | 3.09(1.49,4.70) | **<0.001** | 2.71(1.10,4.33) | | **0.001** |
| General | 1.87(0.37,3.37) | **0.015** | 1.95(0.42,3.48) | | **0.012** |
| Neutral | 3.33(1.81,4.85) | **<0.001** | 3.64(2.05,5.23) | | **<0.001** |
| Tremendous | 3.62(1.44,5.80) | **0.001** | 3.73(1.46,5.99) | | **0.001** |
| **The number of meals you eat every day** |  |  |  | |  |
| 1-2 times |  |  |  | |  |
| 3 times | 1.30(0.42,2.18) | **0.004** | 1.21(0.31,2.11) | | 0.008 |
| 4-5 times | -0.12(-2.80,2.54) | 0.926 | 0.08(-2.55,2.72) | | 0.95 |
| **How much snacks do you eat every day** |  |  |  | |  |
| None |  |  |  | |  |
| Less (1-2 times) | -0.17(-1.22,0.86) | 0.735 |  | |  |
| Neutral (3-4 times) | -0.53(-1.73,0.65) | 0.378 |  | |  |
| Often (5-6 times) | -1.42(-2.88,0.03) | 0.056 |  | |  |
| Always (more than 6 times) | -3.17(-6.71,0.36) | 0.079 |  | |  |
| **Your daily intake of sugary beverages** |  |  |  | |  |
| None |  |  |  | |  |
| Neutral (1-2 times) | 0.33(-0.36,1.03) | 0.35 |  | |  |
| More (more than 3 times) | -0.80(-2.06,0.46) | 0.214 |  | |  |
| **Your average weekly amount of moderate-to-vigorous physical activity:** |  |  |  | |  |
| None |  |  |  | |  |
| 0-150min | 0.30(-0.47,1.07) | 0.443 |  | |  |
| 150-300min | 0.45(-0.58,1.49) | 0.392 |  | |  |
| >300min | -0.50(-2.26,1.25) | 0.571 |  | |  |
| **Your daily alcohol intake** |  |  |  | |  |
| None |  |  |  | |  |
| Less than 15g alcohol (15g alcohol is about 450ml beer or 150ml wine or 50g (1 two) 38 proof liquor or 30g 52 proof liquor) | -0.05(-0.83,0.72) | 0.897 |  | |  |
| More than 15g alcohol (including 15g) | 0.28(-1.27,1.83) | 0.724 |  | |  |
