# Supplementary material for: Knowledge, attitude, and practice towards fatty liver disease among the general population in Shanghai, China: a community-based cross-sectional study
Source: Front Public Health. 2026 May 28;14:1844298. doi: 10.3389/fpubh.2026.1844298 (PMC13255343; doi:10.3389/fpubh.2026.1844298)
Supplement: Supplementary file 4 [file Table_4.docx]

**Suppl 4 Multivariate linear regression analysis of factors associated with practice scores.**

| **Practice** | **Univariate analysis** | | **Multivariate analysis** | |
| --- | --- | --- | --- | --- |
|  | **Coef. (95%CI)** | **P** | **Coef. (95%CI)** | **P** |
| **Knowledge score** | 0.29(0.10,0.48) | **0.002** | 0.08(-0.08,0.24) | 0.331 |
| **Attitude score** | 0.57(0.50,0.64) | **<0.001** | 0.53(0.46,0.60) | **<0.001** |
| **Gender** |  |  |  |  |
| male |  |  |  |  |
| female | 0.59(-0.24,1.44) | 0.166 |  |  |
| **Age (years)** |  |  |  |  |
| <30 |  |  |  |  |
| 31-40 | 2.92(1.94,3.90) | **<0.001** | 0.87(-0.18,1.93) | 0.107 |
| 41-50 | 1.37(0.12,2.62) | **0.032** | 0.23(-1.06,1.53) | 0.72 |
| 51-60 | -0.09(-2.10,1.92) | 0.929 | -0.64(-2.55,1.26) | 0.508 |
| >60 | -1.35(-4.39,1.69) | 0.385 | 0.15(-2.65,2.97) | 0.912 |
| **Marital status** |  |  |  |  |
| Never married |  |  |  |  |
| Married | 3.39(2.49,4.30) | **<0.001** | 1.74(0.69,2.79) | **0.001** |
| Divorced | -2.31(-5.66,1.03) | 0.175 | -1.27(-4.18,1.63) | 0.39 |
| Widowed | 0.38(-5.50,6.27) | 0.899 | 3.62(-1.57,8.81) | 0.172 |
| **Highest degree** |  |  |  |  |
| Junior high school and below |  |  |  |  |
| Senior high school | 1.49(-1.96,4.94) | 0.397 | 1.22(-1.61,4.06) | 0.398 |
| University | 2.76(-0.43,5.96) | 0.09 | 2.00(-0.66,4.67) | 0.14 |
| Postgraduate or above | 3.51(0.15,6.88) | **0.041** | 1.95(-0.85,4.76) | 0.172 |
| **Monthly household income (including physical income, rental income, etc.)** |  |  |  |  |
| <2000 CNY |  |  |  |  |
| 2000-5000 CNY | -1.15(-5.30,3.00) | 0.586 |  |  |
| 5000-10000 CNY | 0.72(-3.21,4.66) | 0.718 |  |  |
| 10000-20000 CNY | 1.70(-2.20,5.61) | 0.393 |  |  |
| >20000 CNY | 2.54(-1.37,6.46) | 0.203 |  |  |
| **Occupation type** |  |  |  |  |
| Leader of governemental organizations, enterprises or institutions |  |  |  |  |
| Professional and technical personnel (teachers, doctors, engineering and technical personnel, writers and other professionals) | -2.75(-4.26,-1.23) | **<0.001** | -2.36(-3.66,-1.06) | **<0.001** |
| Office staff and related personnel | -4.10(-5.67,-2.54) | **<0.001** | -2.86(-4.20,-1.53) | **<0.001** |
| Business, service personnel | -5.00(-6.65,-3.36) | **<0.001** | -3.21(-4.62,-1.80) | **<0.001** |
| Agricultural, forestry, animal husbandry, fishery water conservancy production personnel | -3.18(-6.88,0.51) | 0.092 | -0.75(-3.95,2.44) | 0.645 |
| Production, transportation equipment operators and related personnel | -4.03(-6.29,-1.78) | **<0.001** | -2.96(-4.87,-1.04) | **0.002** |
| Military personnel | 0.96(-8.29,10.2) | 0.839 | -0.08(-7.85,7.68) | 0.984 |
| Other | -6.66(-8.24,-5.07) | **<0.001** | -3.73(-5.19,-2.28) | **<0.001** |
| **BMI** | -0.05(-0.18,0.07) | 0.415 |  |  |
| **Sleep quality** |  |  |  |  |
| Very good |  |  |  |  |
| Good | -1.03(-2.19,0.13) | 0.082 | -0.68(-1.66,0.29) | 0.173 |
| Neutral | -3.40(-4.61,-2.18) | **<0.001** | -1.98(-3.02,-0.94) | **<0.001** |
| Poor | -3.56(-5.28,-1.83) | **<0.001** | -1.60(-3.06,-0.15) | **0.03** |
| Very poor | -4.03(-8.80,0.72) | 0.096 | -4.28(-8.27,-0.30) | **0.035** |
| **How stressful you feel about your daily life and work** |  |  |  |  |
| Rarely |  |  |  |  |
| Less | 2.04(-0.08,4.17) | 0.06 |  |  |
| General | 0.22(-1.76,2.21) | 0.824 |  |  |
| Neutral | 0.14(-1.86,2.16) | 0.886 |  |  |
| Tremendous | 0.14(-2.75,3.03) | 0.924 |  |  |
| **The number of meals you eat every day** |  |  |  |  |
| 1-2 times |  |  |  |  |
| 3 times | 2.35(1.20,3.50) | **<0.001** | -0.10(-1.09,0.89) | 0.842 |
| 4-5 times | 2.06(-1.43,5.57) | 0.246 | 0.03(-2.90,2.97) | 0.983 |
| **How much snacks do you eat every day** |  |  |  |  |
| None |  |  |  |  |
| Less (1-2 times) | 0.02(-1.33,1.39) | 0.967 | 0.13(-0.97,1.25) | 0.807 |
| Neutral (3-4 times) | -0.19(-1.76,1.37) | 0.808 | 0.08(-1.21,1.38) | 0.9 |
| Often (5-6 times) | -0.04(-1.96,1.87) | 0.961 | 1.26(-0.31,2.84) | 0.116 |
| Always (more than 6 times) | -5.38(-10.0,-0.73) | **0.023** | -2.64(-6.48,1.19) | 0.177 |
| **Your daily intake of sugary beverages** |  |  |  |  |
| None |  |  |  |  |
| Neutral (1-2 times) | -0.22(-1.14,0.69) | 0.637 |  |  |
| More (more than 3 times) | -0.02(-1.69,1.64) | 0.976 |  |  |
| **Your average weekly amount of moderate-to-vigorous physical activity** |  |  |  |  |
| None |  |  |  |  |
| 0-150 min | 4.30(3.34,5.26) | **<0.001** | 3.38(2.53,4.22) | **<0.001** |
| 150-300 min | 6.20(4.91,7.49) | **<0.001** | 4.25(3.08,5.43) | **<0.001** |
| >300 min | 5.97(3.78,8.16) | **<0.001** | 5.16(3.22,7.10) | **<0.001** |
| **Your daily alcohol intake** |  |  |  |  |
| None |  |  |  |  |
| Less than 15g alcohol (15g alcohol is about 450ml beer or 150ml wine or 50g (1 two) 38 proof liquor or 30g 52 proof liquor) | 2.14(1.13,3.16) | **<0.001** | 1.01(0.15,1.87) | **0.021** |
| More than 15g alcohol (including 15g) | 1.18(-0.84,3.21) | 0.252 | -0.73(-2.43,0.96) | 0.397 |
